# A Tetravalent Biparatopic Antibody Causes Strong HER2 Internalization and Inhibits Cellular Proliferation

**DOI:** 10.3390/life11111157

**Published:** 2021-10-29

**Authors:** Filippo Benedetti, Katharina Stadlbauer, Gerhard Stadlmayr, Florian Rüker, Gordana Wozniak-Knopp

**Affiliations:** Christian Doppler Laboratory for Innovative Immunotherapeutics, Institute of Molecular Biotechnology, Department of Biotechnology, University of Natural Resources and Life Sciences, Muthgasse 18, 1190 Vienna, Austria; Filippo.benedetti@boku.ac.at (F.B.); katharina.stadlbauer@boku.ac.at (K.S.); Gerhard.stadlmayr@boku.ac.at (G.S.); florian.rueker@boku.ac.at (F.R.)

**Keywords:** biparatopic targeting, bispecific antibodies, Fcab, HER2 internalization, HER2 overexpression, mAb^2^, pertuzumab, trastuzumab

## Abstract

The overexpression of tyrosine kinase HER2 in numerous cancers, connected with fierce signaling and uncontrolled proliferation, makes it a suitable target for immunotherapy. The acquisition of resistance to currently used compounds and the multiplicity of signaling pathways involved prompted research into the discovery of novel binders as well as treatment options with multiple targeting and multispecific agents. Here we constructed an anti-HER2 tetravalent and biparatopic symmetrical IgG-like molecule by combining the Fab of pertuzumab with a HER2-specific Fcab (Fc fragment with antigen binding), which recognizes an epitope overlapping with trastuzumab. In the strongly HER2-positive cell line SK-BR-3, the molecule induced a rapid and efficient reduction in surface HER2 levels. A potent anti-proliferative effect, specific for the HER2-positive cell line, was observed in vitro, following the induction of apoptosis, and this could not be achieved with treatment with the mixture of pertuzumab and the parental Fcab. The inhibitory cytotoxic effect of our antibody as a single agent makes it a promising contribution to the armory of anti-cancer molecules directed against HER2-addicted cells.

## 1. Introduction

In numerous forms of cancerous disease, HER2-overexpressing tumors present a large proportion of diagnosed cases, notably in 15–20% of breast cancer [1], 22% of gastric tumors [2], and 4% of colorectal tumors [3], and HER2 overexpression is typically connected with poor prognosis. The overamplification of the Her2 gene in these cells causes the expression of large numbers of HER2 receptors on the cell surface, leading to potent proliferation after the induction of tyrosine kinase signaling [4] caused by the homodimerization of HER2 [5] and heterodimerization of HER2 with other HER-family members [6]. Apart from proliferation stimulation, HER2 overexpression also promotes the epithelial to mesenchymal transition of tumor cells, which is accompanied with an enhanced expression of metalloproteinase, causing HER2 shedding and the accumulation of intracellular carboxy-terminal HER2 component p95, a predictive marker for trastuzumab resistance [7], as well as the degradation of extracellular matrix proteins and the concomitant increase in tumor cell migration, which enables rapid invasion [8]. At the same time, HER2 expression on tumors can lead to evasion from the immune response with the emergence of escape variants showing a down-regulation of MHC class I [9] and impairment of the T-cell recognition of HLA-A2 restricted antigens [10]. Collectively, the proliferation ability of such tumors is so strongly dependent on HER2 that it can be considered a “HER2 addiction” [11], and this has led to the development of anti-HER2 therapies dramatically improving the treatment options for patients with HER2-positive cancers [12].

At the molecular level, the extracellular domain (ECD) of HER-family members is composed of four subdomains labeled from I to IV with IV being the most proximal to the cellular membrane. Only the extended (open) form of the ECD, supported by the interactions of globular subdomains I and III, is signaling-competent as opposed to the tethered form where rod-like cysteine-rich subdomains II and IV are strongly interacting [13]. For HER2, only the open conformation has been observed [14]. Currently approved therapeutic approaches specifically addressing HER2-positive cells include many monoclonal antibodies and their variants. Trastuzumab (Herceptin) [15], which binds to subdomain IV, acts primarily via antibody-dependent cytotoxicity, but causes also the downregulation of surface levels of HER2, inhibits proliferation directly via the induction of cell cycle arrest and prevents the cleavage of the p95 component [16]. Its derivate trastuzumab T-DM1 (trastuzumab emtansin, Kadcyla) is an antibody-drug conjugate (ADC) that after the internalization and release of the toxin component causes apoptosis of target cells [17]; recently, two further ADCs based on trastuzumab were approved for clinical use [18]. Pertuzumab (Perjeta) targeting HER2 subdomain II prevents dimerization and subsequent tyrosine-kinase mediated signaling [13]. The clinical study Cleopatra (NCT00567190) including 1196 patients dosed between 2008 and 2011, performed by Hoffmann-La Roche and Genentech, proved a significant beneficial effect of the simultaneous application of trastuzumab and pertuzumab for patients with HER2-positive metastatic breast cancer, with an improvement in overall survival with pertuzumab, trastuzumab, and docetaxel versus a placebo, trastuzumab, and docetaxel maintained after a median of more than 8 years of follow-up [19]. Pertuzumab also enhanced the anti-tumor activity of T-DM1 in mouse xenograft models [20]. This prompted research into the use of bispecific and multispecific constructs, able of targeting HER2 at different epitopes with multiple possible valencies.

Early attempts of the multispecific targeting of HER2 involved the combination of variable domains of trastuzumab and pertuzumab into a tetravalent bispecific dual-variable-domain antibody-like fusion protein, which showed a potentiated ability of the inhibition of the growth of HER2-positive cell lines [21]. In another study, variable domains of trastuzumab or pertuzumab were fused with short peptide linkers to each other in various constellations, and one fusion variant retained the full binding activities of both parental antibodies and prevented HER2 heterodimerization more potently than their combination [22]. A bispecific antibody with one trastuzumab- and one pertuzumab-based Fab arm was constructed with “knobs-into-holes” based heterodimerization and post-expression assembly and could inhibit the proliferation of HER2-positive breast cancer cells; however, the anti-tumor activity of the bispecific construct was similar to the combination of trastuzumab and pertuzumab [23]. Recently, a fusion construct of trastuzumab and two single-chain antibodies targeting the HER2 subdomain I was described to exhibit an antiproliferative activity beyond the parental antibodies, caused by HER2 clustering into inactive complexes, followed by their robust internalization and degradation [24]. Not only antibodies, also alternative binding scaffolds were successfully used for biparatopic targeting: a fusion protein of two ankyrines targeting subdomain I and subdomain IV of HER2 was able of the strong inhibition of the proliferation of HER2-positive cells with the proposed mechanism of changing the conformation of the extracellular domain of HER2 in a handcuff-like manner and the inhibition of HER2-mediated signaling [25].

As the multivalent and multiparatopic targeting of HER2 appears a compelling way of the inhibition of the proliferation of HER2-positive cells, we designed a symmetrical bispecific construct based on a monoclonal antibody with a novel antigen-binding site in the C_H_3 domains; we used the pertuzumab scaffold with an anti-HER2 Fc antigen-binding fragment (Fcab). The chosen Fcab binds to an epitope that overlaps with the a trastuzumab on subdomain IV of HER2 [26,27] and is able of bivalent binding [27]. Three therapeutic candidate molecules of the described bispecific format, mAb^2^, have already entered the stage of clinical testing [28,29,30].

## 2. Materials and Methods

### 2.1. Expression Constructs

Sequences of pertuzumab were cloned into the pTT5 vector (Canadian National Research Council, CNRC, Ottawa, ON, Canada). The affinity matured anti-HER2 reactive Fcab 3_17 was isolated in the selection campaigns along with the well-characterized clone H10-3-6 [26,31,32], carrying the unexpected scaffold mutation S324T, and was modified with the mutation R415bE (2nd inserted residue after the position 415) (EU residue numbering [33] and amino acid residues inserted C-terminally from the position 415 are labelled a–e), which was discovered to enhance its physicochemical properties [34]. For mutagenesis, the QuikChange Lightning Site-Directed Mutagenesis Kit (Agilent Technologies, Santa Clara, CA, USA) was used with oligonucleotides 3_17E and 3_17Ea (oligonucleotide sequences in Supplementary Table S1) exactly according to the manufacturer’s instructions. The C_H_3 domain of the Fcab was amplified with PCR with oligonucleotides ch3xho1 and ch3sbam2, cut with restriction enzymes *Bsr*GI and *Bam*HI (New England Biolabs, Ipswich, MA, USA) and ligated with the vector containing a cloned pertuzumab heavy chain sequence excluding the wild-type C_H_3 domain sequence (sequences of all constructs in the Supplementary Table S2). Plasmids were transformed, amplified in *E. coli* TOP10 (Thermo Fisher Scientific, Waltham, MA, USA), and isolated using midipreparation (Macherey-Nagel, Düren, Germany).

### 2.2. Protein Production

Polyethyleneimine (PEI)-mediated transient transfection of HEK293-6E cells (CNRC, Ottawa, ON, Canada) was performed by first combining the plasmids encoding the heavy and light chain in a 1:1 mass ratio, and incubating them for 15 min at room temperature (RT) with double mass of PEI. The mix containing 1 µg total DNA /mL culture was delivered dropwise to the cells at a density of 1.5–2.0 × 10^6^ /mL, and the expression continued at 37 °C in a humidified atmosphere with 5% CO_2_. Two days post transfection, cells were fed with tryptone TN-20 to a final concentration of 0.5%, and a supernatant was harvested 5 days post transfection with centrifugation at 1000× *g*, 20 min at 4 °C. A 1 M Na-phosphate stock buffer, pH 7.0, was added to the clarified supernatants to a 0.1 M final concentration, and samples were filtered through a 0.45-µm filter before loading onto a Protein A HP column (Cytiva, Marlborough, MA, USA), equilibrated with the 0.1 M Na-phosphate buffer, pH 7.0. Unbound material was washed away with the same buffer and antibodies were eluted with 0.1 M glycine, pH 3.5. Fractions were neutralized immediately and dialyzed against a 100-fold volume of phosphate-buffered saline (PBS) overnight at 4 °C using Snakeskin dialysis tubing with a 10,000 Da molecular weight cut-off (MWCO) (Pierce, Thermo Fisher Scientific, Waltham, MA, USA). The proteins were stored at −80 °C until use. Trastuzumab, trastuzumab-CT6 mAb^2^ [35], and control Fcab constructs (wild-type Fc and 3-17ET) were prepared in the same way. VEGF was produced in HEK293-6E cells and purified as described previously [36].

### 2.3. Labelling of Antibody Preparations

Direct labeling of pertuzumab and pertuzumab-based mAb^2^ was performed using the Alexa Fluor 488 Protein labeling kit (Thermo Fisher Scientific, Waltham, MA, USA), exactly according to the manufacturer’s instructions.

Direct labeling of 9G6 antibody for microscopy experiments was done with the Alexa Fluor 488 Zenon Mouse IgG_1_ Labeling Kit (Invitrogen, Thermo Fisher Scientific, Waltham, MA, USA) exactly according to the manufacturer’s instructions.

For monitoring the internalization of pertuzumab, the biparatopic antibody, and the constructs used as a positive control for the internalization of trastuzumab and trastuzumab-CT6, preparations were labeled with CypHer5E Mono NHS Ester (Cytiva, Marlborough, MA, USA) at 0.5 mg/mL in 10:1 dye to a protein molar ratio for 1 h at room temperature and subsequently dialyzed against a 100-fold volume of PBS overnight at 4 °C in the dark using Snakeskin dialysis tubing with 10000 Da MWCO (Pierce, Thermo Fisher Scientific, Waltham, MA, USA).

### 2.4. High Performance Liquid Chromatography—Size Exclusion Chromatography (HPLC-SEC)

The Shimadzu LC-20A Prominence system equipped with a diode array detector was used to perform HPLC-SEC with a Superdex 200 Increase 10/300 GL column (Cytiva, Marlborough, MA, USA). The mobile phase buffer used was PBS with 200 mM NaCl. Chromatography was conducted with a constant flow rate of 0.75 mL/min. A total of 20 µg protein at about 1 mg/mL were loaded on the column for analysis. Column calibration was performed with a set of molecular weight standards ranging from 1.3 to 670 kDa (Bio-Rad, Hercules, CA, USA).

### 2.5. Cell Culture and Cell Staining

SK-BR-3 (ATCC-HTB30) and MD-MBA-468 (ATCC HTB-132) cell lines were obtained from ATCC and kept in Dulbecco’s modified Eagle Medium (DMEM) (Biochrom, Berlin, Germany) with 10% FBS (Biochrom) and penicillin-streptomycin (Gibco/Thermo Fisher Scientific, Waltham, MA, USA) in a humidified atmosphere at 37 °C under 5% CO_2_ and passaged routinely twice a week. Cells were harvested with a Biotase detachment reagent (Biochrom, Berlin, Germany) and resuspended to a density of 1 × 10^6^ cells/mL for blocking with 2% bovine serum albumin (BSA)-PBS for 30 min on ice. Then, 100-µL-aliquots were distributed into the wells of a 96-well-plate. After centrifugation for 5 min at 300× *g* and 4 °C, the cells were stained with a 3-fold serial dilution of pertuzumab starting from 66.6 nM in 2% BSA-PBS for 30 min on ice in duplicates. A 1:100 dilution of anti-human kappa chain-fluorescein isothiocyanate (FITC) conjugate (Cat.No. F-3761, Sigma-Aldrich, St. Louis, MO, USA) in 2% BSA-PBS was used to detect test protein binding. Cells were finally resuspended in 200 µL ice-cold PBS with 7-aminoactinomyin (Becton Dickinson, Franklin Lakes, NJ, USA) and analyzed with a Gallios flow cytometer (Beckman Coulter, Brea, CA, USA). Cetuximab (Erbitux®) (Merck KGaA, Darmstadt, Germany) was purchased from the pharmacy.

Binding of 3_17ET Fcab was determined after incubation with 3-fold serial dilutions starting from 333 nM in 2% BSA-PBS for 3 min on ice in duplicates. A 1:1000 dilution of phycoerythrin-labeled anti-human IgG (γ-chain specific), F(ab′)_2_ fragment (Cat. No. P-8047, Sigma-Aldrich, St. Louis, MO, USA) was used for detection with a Guava EasyCyte Flow Cytometer (Luminex, Austin, TX, USA). Data on sample fluorescence were processed using Kaluza software to obtain the geometric mean of cell fluorescence. To examine the binding to the SK-BR-3 cell surface that may differ between the pertuzumab and biparatopic antibody, cell aliquots were incubated with 66.6 nM pertuzumab or 100 nM 3_17ET Fcab in 100 µL 2% BSA-PBS for 30 min on ice after blocking, and then stained with graded concentrations of the bispecific antibody, directly labeled with Alexa Fluor 488, in 3-fold dilution steps starting at 66.6 nM.

For the evaluation of HER2 surface levels, the SK-BR-3 cells were seeded into 12-well plates at a density of 100,000 cells/well and allowed to attach overnight. Then they were treated for the indicated period of time with a 66.6 nM solution of the biparatopic antibody, a mixture of pertuzumab and 3_17ET Fcab or a mixture of the IgG1-kappa isotype control and wild-type Fc. Untreated cells were used as a control. After harvesting with a cell dissociation reagent (Gibco, Thermo Fisher Scientific, Waltham, MA, USA), the cells were resuspended in the cell culture medium with 10% FBS. Cells were blocked with 2% BSA-PBS for 30 min on ice and stained for the surface levels of HER2 with the anti-HER2 antibody 9G6 (Cat#sc-08; Santa Cruz, Dallas, Texas, RRID: AB_627998), recognizing a distinct epitope of pertuzumab and trastuzumab [37], at 2.5 µg/mL in 2% BSA-PBS for 30 min on ice in a 100-µL volume. Its binding was detected with an anti-mouse-(Fab)’_2_—FITC conjugate (Cat.No. F-2653, Sigma-Aldrich, St. Louis, MO, USA), used at a 1:200 dilution in 2% BSA-PBS for 30 min on ice in a 100-µL volume. After resuspending the cells in 200 µL ice-cold PBS, fluorescence levels were recorded with a Guava EasyCyte Flow Cytometer (Luminex, Austin, TX, USA) and expressed as the % fluorescence of untreated cells. Duplicate measurements were taken.

Cytotoxicity assay. Antigen-positive cells and control cells were seeded at a density of 10,000 cells per well into 60 inner wells of a 96-F-shaped-well plate in 100 µL DMEM with 10% FCS and antibiotics, sealed with a Snake-skin semi-permeable membrane (Sigma-Aldrich, St. Louis, MO, USA) and allowed to attach overnight at 37 °C. The treatment proceeded with antibodies in 3-fold-dilutions from 30 to 0.05 nM in the same medium in a 100-µL-volume per well. After 7 days, the culture medium was removed and 100 µL of WST-1 reagent (Roche, Basel, Switzerland), diluted 1:10 in DMEM with 10% FCS with antibiotics, were added to the microtiter plate wells. The absorbance at 450/620 nm was read out after 2–4 h of incubation at 37 °C under a humidified atmosphere with 5% CO_2_. Readings of wells without cells were considered background, and those of wells with cells cultured in the medium without added antibodies served as the untreated control values. All readings were recorded at least in duplicates. Percent survival was calculated as ((OD_450/620_) _test wells_ − (OD_450/620_) _background_)/(OD_450/620_) _untreated control_ − (OD_450/620_) _background_)) × 100, and the EC_50_ was calculated using Prism5. The isotype IgG1-kappa antibody (Cat. No. I5154) was purchased from Sigma-Aldrich (St. Louis, MO, USA).

### 2.6. Fluorescence Microscopy

Detection of surface levels of HER2. SK-BR-3 cells were seeded into 8-well chamber slides (µ-Slide, IBIDI-Treat surface, IBIDI, Gräfelfing, Germany) with lysine coating and treated for 48 h with bispecific PE_3_17ET, a mixture of PE, and 3_17ET Fcab or a mixture of the IgG1-kappa isotype and wild-type Fc fragment. The next day, cells were rinsed with 2 consecutive washes with PBS and then stained with 2.5 µg/mL 9G6 antibody, complexed with the Alexa Fluor 488 Zenon Mouse IgG_1_ reagent, for 30 min on ice. The wells were washed twice with PBS and incubated for 5 min at RT in a solution of Hoechst 33342 (Bio-Rad, Hercules, CA, USA), diluted to 1 µg/mL, rinsed twice with PBS, and analyzed with a Leica DMI6000B microscope using HCX Objective Plan-Apochromat 63x/1.4 Oil (Leica Microsystems, Wetzlar, Germany). Data on sample fluorescence were processed using Leica Application Suite X software, version 3.7.0.

Detection of CypHer-5E labelled antibodies. SK-BR-3 cells were seeded in slides as described above and then treated with antibodies diluted in the culture medium with 10% FBS and antibiotics at 37 °C, under 5% CO_2_ in a humidified atmosphere. Cells were imaged at different time points as indicated in the Results section. Pertuzumab and pertuzumab-mAb^2^ were used at 66.7 nM and trastuzumab and trastuzumab-mAb^2^ at 33.3 nM; VEGF as a cross-linker was added at a 1 µM concentration. Before imaging, the medium was removed, the cells washed briefly 3 times with PBS, stained with 1 µg/mL Hoechst 33342 (Bio-Rad, Hercules, CA, USA) for 5 min at RT, and washed twice with PBS. Confocal microscopy was performed on a Leica TCS SP5 spot scanning confocal microscope equipped with a HCX PL APO CS 40x/0.85 dry objective, HyD detector, and Argon-laser (Leica Microsystems, Wetzlar, Germany). Cropping, brightness, and contrast adjustments, as well as the evaluation of the fluorescence of individual cells (where brightness and contrast adjustments were not applied) were conducted with Image J (Rasband W.S. ImageJ, U.S. National Institutes of Health, Bethesda, MD, USA). The corrected total cell fluorescence (CTCF) for 10 individual cells per sample was determined using the equation CTCF = Integrated Density − (area of selected cell × mean fluorescence of background readings).

Apoptosis assays. SK-BR3 cells were seeded at 100,000 cells/well in 1 mL DMEM with 10% FCS and antibiotics into the wells of a 12-well-plate and were allowed to attach overnight at 37 °C, under 5% CO_2_ in a humidified atmosphere. After incubation with 20 nM antibodies for 36 or 96 h, cells were harvested: detached cells from the culture supernatant as well as dissociation reagent detached cells were pelleted with a centrifugation step at 300× *g*, 4 °C for 5 min. For annexinV staining, the cells were resuspended in 1 mL of the AnnexinV-binding buffer (Thermo Fisher Scientific, Waltham, MA, USA) containing 10 µL of the AnnexinV-Alexa Fluor 647 reagent (Thermo Fisher Scientific, Waltham, MA, USA) and 1 µg/mL propidium iodide (PI) (Sigma-Aldrich, St. Louis, MO, USA). After incubation for 15 min at RT, cells were analyzed using the Guava EasyCyte Flow Cytometer (Luminex, Austin, TX, USA). Only AnnexinV-positive cells were considered early apoptotic and AnnexinV, as well as PI-positive cells were considered late apoptotic. For the detection of Caspase 3/7 activity, the cells were resuspended in 100 µL of PBS with the addition of 2 droplets/mL of the Cell EventTM Caspase-3/7 Green Ready Probes reagent (Thermo Fisher Scientific, Waltham, MA, USA) and incubated for 45 min at RT. To each sample, 300 µL of PBS and PI to 1 µg/mL of the final concentration were added. After incubation for 15 min at RT, cells were analyzed using the Guava EasyCyte Flow Cytometer (LuminexAustin, TX, USA). Only Caspase 3/7-positive cells were considered early apoptotic, and Caspase 3/7 as well as PI-positive cells were considered late apoptotic. All measurements were conducted in duplicates.

## 3. Results

### 3.1. Expression of the Bispecific Antibody

**Figure 1:**
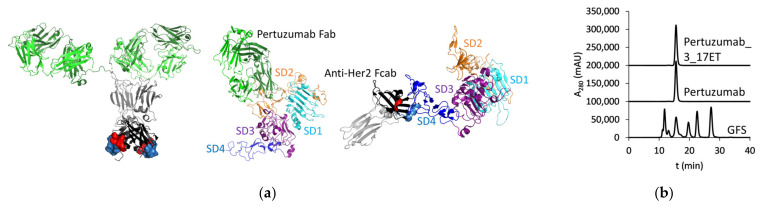
Structure of mAb^2^. (**a**) Left panel: Cartoon diagram of a biparatopic mAb^2^ antibody composed of Pertuzumab Fab (dark green: V_H_-C_H_1, light green: V_L_-C_L_) and antigen-binding Fc (Fcab) (gray: C_H_2, black: C_H_3, red: residues mutated in AB loop, light blue: residues mutated in EF loop) (PDB: 1HZH), central panel: Pertuzumab Fab in complex with HER2 extracellular domain (ECD) (PDB: 1S78), right panel: 3_17ET-related Fcab in complex with HER2 ECD (PDB: 5K33) (elements of Fab and Fcab are colored as described above, SD—subdomain). Figure was prepared using The PyMOL Molecular Graphics System, Schrödinger, LLC; (**b**) HPLC-SEC of pertuzumab and pertuzumab-based mAb^2^. Gel filtration standard (GFS) included 670, 158, 44, 17, and 1.35 kDa-proteins (Bio-Rad).

We constructed a bispecific antibody composed of variable regions of pertuzumab and an Fc-fragment with a novel binding site for HER2 (Figure 1A, left panel), binding on one hand to subdomain II via pertuzumab arms and on the other hand to subdomain IV via the modified C_H_3 domain (Figure 1A, right panel). This molecule could be expressed at levels similar to pertuzumab (5.8 mg/L vs. 16 mg/L) and exhibited a monomeric profile in HPLC-SEC with an elution time identical to the parental antibody (Figure 1B). The Fcab 3_17ET used as a building block of the studied bispecific antibody recognizes HER2 with a K_D_ of 2.29 × 10^−8^ M (Supplementary Figure S1A) and exhibits a monomeric profile in HPLC-SEC also as a stand-alone protein (Supplementary Figure S1B) (description of methods in Supplementary File). Pertuzumab binds to HER2 with about 1.5 × 10^−8^ M affinity [38].

### 3.2. Cell Surface Binding

**Figure 2:**
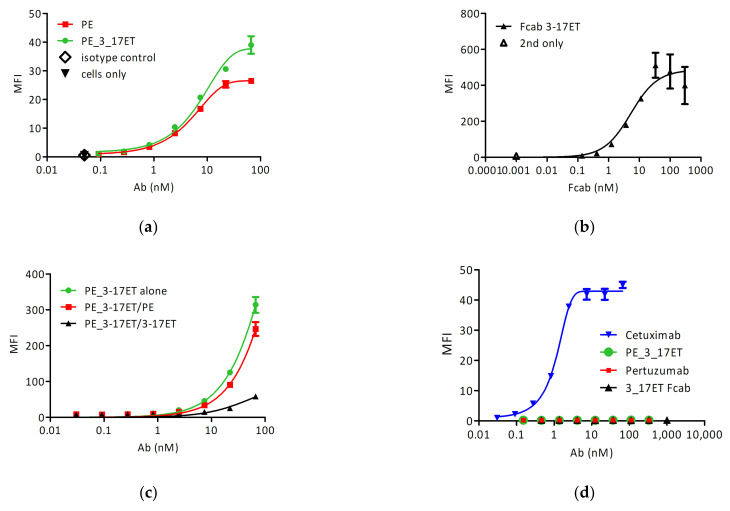
Cell surface staining with pertuzumab and pertuzumab_3_17ET. (**a**) Staining of SK-BR-3 with directly labelled pertuzumab and pertuzumab-3_17ET; (**b**) Staining of SK-BR-3 with 3_17 Fcab; (**c**) Staining of SK-BR-3 with bispecific antibody after saturation with pertuzumab or 3_17ET Fcab (**d**) Staining of the control cell line MDA-MB-468 with the anti-HER1 antibody cetuximab (positive control), pertuzumab, 3_17ET Fcab, and the bispecific antibody.

First, we examined the ability of the bispecific antibody to bind to the surface of strongly HER2-positive SK-BR-3 cells. We determined the saturation of binding with pertuzumab at approximately 66 nM with an EC_50_ of 9.9 nM (Figure 2A). The 3_17 ET Fcab saturated the surface of SK-BR-3 cells at about 100 nM (Figure 2B). We then incubated the cells with a saturation concentration of pertuzumab and performed the staining with a dilution series of directly labeled preparations of the wild-type and the biparatopic antibody. While we could observe a signal upon staining with the biparatopic antibody, no reactivity of SK-BR-3 cells with the directly labeled wild-type antibody could be detected, indicating that the binding of the bispecific antibody proceeded through a different epitope of HER2 (Figure 2C). The same competition experiment with the bispecific antibody was performed after the saturation of the cell surface with 3_17 Fcab (Figure 2C). The bispecific construct as well as the controls did not display any reactivity with HER2 negative MDA-MB-468 cells (Figure 2D), where cetuximab was used as a positive control for HER1 expression.

### 3.3. Effect of the Bispecific Antibody on HER2-Overexpressing Cells

**Figure 3:**
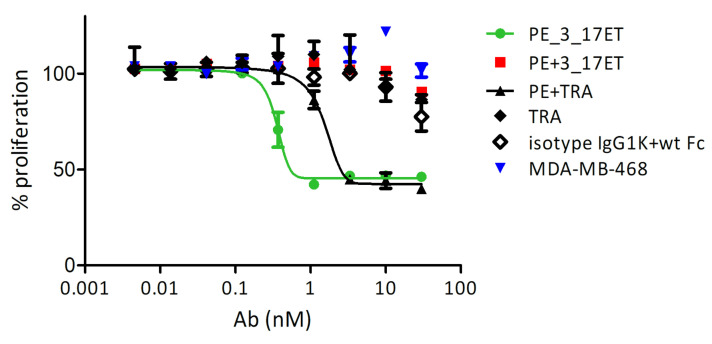
Proliferation assay on SK-BR-3 cells demonstrates the activity of bispecific construct for HER2-positive cells which were treated with pertuzumab_3_17ET, pertuzumab and 3_17ET Fcab, trastuzumab and pertuzumab, trastuzumab alone, orhuman IgG1 kappa isotype, and wild-type (wt) Fc. Blue triangles present the effect of pertuzumab_3_17ET bispecific antibody on MDA-MB-468 cells.

We were further interested if the treatment of SK-BR-3 cells with the biparatopic antibody has an effect on their proliferation. While the proliferation of the cells incubated with a mixture of pertuzumab and 3_17ET Fcab or with trastuzumab alone was not lower than for cells incubated with the mix of the isotype antibody and wild-type Fc, the biparatopic antibody induced a strong anti-proliferative effect with an EC_50_ of about 0.3 nM (Figure 3). The same construct had no effect on the antigen-negative MDA-MB-468 cells. When SK-BR-3 cells were treated with the combination of pertuzumab and trastuzumab, their proliferation was strongly inhibited, but the EC_50_ of the biparatopic construct in the same experiment was 10-fold lower (Figure 3).

### 3.4. Bispecific Antibody Depletes HER2 from the Cell Surface

**Figure 4:**
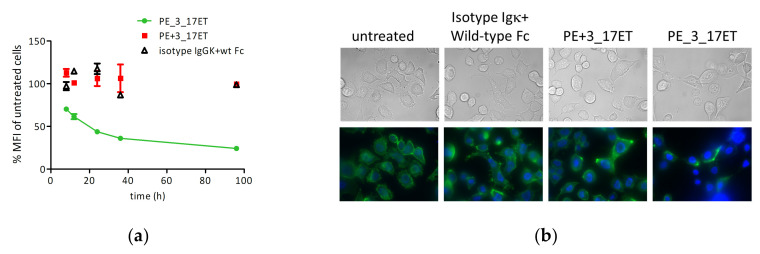
HER2 surface levels decrease after treatment with pertuzumab_3_17ET bispecific antibody. (**a**) HER2 surface levels monitored with 9G6 antibody binding in FACS after 8–96h of treatment with pertuzumab_3_17ET, mixture of pertuzumab, and 3_17 Fcab or isotype controls; (**b**) immunofluorescence of SKBR3 cells, untreated or treated with isotype controls IgG1-kappa and wild-type Fc, mixture of pertuzumab, and 3_17ET Fcab or pertuzumab_3_17ET, for 48 h. Cells were stained with labelled 9G6 antibody (green fluorescence) and counterstained with Hoechst 33342 (blue fluorescence). MFI, mean fluorescence intensity.

To elucidate the mechanism of proliferation inhibition, the surface level of HER2 after several hours of the incubation of SK-BR-3 cells with the biparatopic construct were measured using an antibody recognizing an epitope different from pertuzumab or trastuzumab. The levels of HER2 gradually receded from the timepoint of 8 h and reached a minimum after 36 h (Figure 4A). The microscopic observation of cellular immunofluorescence confirmed the absence of HER2 from the surface of treated cells (Figure 4B).

**Figure 5:**
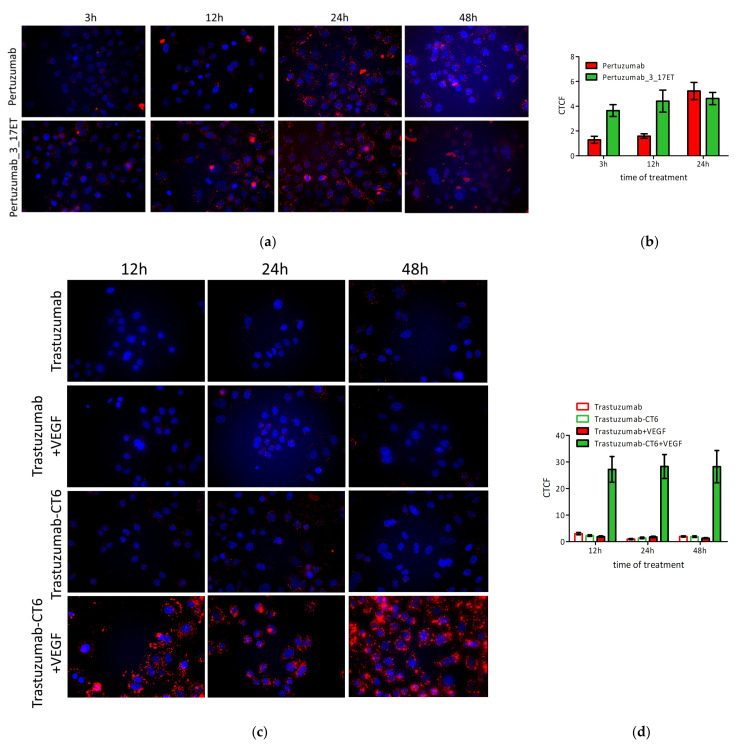
Internalization of anti-HER2 antibodies followed by CypHer5 fluorescence. Red fluorescence is the signal from CypHer5 and blue from Hoechst 33342 (nucleus staining). (**a**) SK-BR-3 cells treated with pertuzumab-CypHer5 and pertuzumab_3_17ET-CypHer5 for 3, 12, 24, and 48 h; (**b**) CTCF of pertuzumab-CypHer5 and pertuzumab_3_17ET-CypHer5 treated cells; (**c**) SK-BR-3 cells treated with trastuzumab-CypHer5 and trastuzumab-CT6-CypHer5 for 12, 24, and 48 h; (**d**) CTCF of trastuzumab-CypHer5, trastuzumab-CT6-CypHer5, trastuzumab-CypHer5, and VEGF, trastuzumab-CT6-CypHer5 and VEGF-treated cells.

The biparatopic construct internalized into the HER2-positive cells more rapidly than the parental antibody, as we have observed 2.8-fold more CTCF after 3 h and after 12 h of treatment (Figure 5A,B). The internalization of the biparatopic antibody could only have been recorded until 24 h after treatment, as after 48 h the cells were disintegrated to a level that rendered imaging impossible (Figure 5A), which also suggests that the effect of the biparatopic antibody was cytotoxic rather than cytostatic. In comparison with the forced internalization into lysosomes of an anti-HER2/anti-VEGF mAb^2^ construct that was used as a positive control (trastuzumab with an anti-VEGF Fcab CT6 [36], when the cross-linking with the dimeric antigen VEGF was performed (Figure 5C,D), the CTCF levels were about 5-fold lower.

### 3.5. Induction of Apoptosis As a Reason for Anti-Proliferative Activity of the Bispecific Antibody

**Figure 6:**
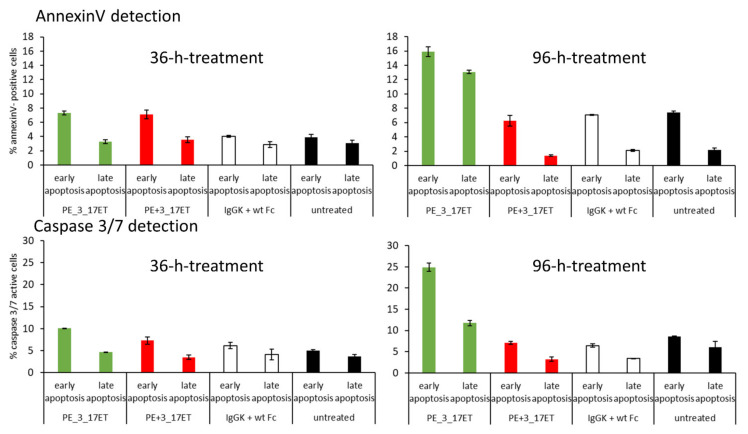
Bispecific antibody induced apoptosis in HER2-positive cells. Detection of annexinV-positive cells and caspase 3/7-active cells after 36 and 96 h of treatment with bispecific antibody pertuzumab (PE)_3_17ET, mixture of PE and 3_17ET, isotype controls IgG1-kappa antibody and wild-type (wt) Fc, and untreated cells. After 96 h, higher numbers of apoptotic cells can be detected after treatment with bispecific antibody compared with the controls.

To find the reason of proliferation inhibition, the levels of apoptosis in treated SK-BR-3 cells were examined after 36 h and 96 h of incubation, using either the annexinV-positive staining or the caspase 3/7 activity as an apoptosis marker (Figure 6). After 36 h of incubation, about 7% of early apoptotic cells could be detected via staining with annexinV upon incubation with the bispecific antibody and mix of pertuzumab and 3_17 Fcab, in comparison to about 4% present in control samples, and below 4% late apoptotic cells were present in all samples. At this timepoint, 10% of the cells treated with the bispecific antibody were detected to be early apoptotic judging from caspase 3/7 activity, 8% of the cells in the sample treated with the mixture of pertuzumab and 3_17ET Fcab, and around 5% in the other two control samples. Again, around 4% late apoptotic cells were detected. After 96 h, there were many more apoptotic cells present in the samples treated with the bispecific antibody: when using annexinV as a marker, 16% of cells were found to be early apoptotic and 13% late apoptotic, in comparison with about 7% and 2–5% found for the control samples. Early apoptotic Caspase 3/7-active cells were at 25% and late apoptotic at 13%, while in all control samples the early apoptotic cells were around 7% and late apoptotic at 2–7%.

## 4. Discussion

The simultaneous engagement of multiple epitopes of the extracellular domain of HER2 appears an attractive option to extend the functionalities of therapeutic antibodies and other binders reactive with a single epitope. Here the mAb^2^ format with a bispecific and tetravalent binding was applied with the introduction of a subdomain IV-reactive Fcab in the scaffold of subdomain II-binding pertuzumab. Its strong cytotoxic effect on HER2-positive cells was not seen upon the treatment with the combination of both agents, indicating that the observed activity is confined to the biparatopic antibody. When antibodies pertuzumab and trastuzumab were used in combination, the reduction in cell proliferation was less potent than with the bispecific antibody. It was observed previously that the treatment of HER2-positive cells with pertuzumab, trastuzumab, or their combination reduced the fraction of proliferating cells in vitro [39], but not that they could elicit a potent elimination of HER2 from the cell surface, the effect we observed with the biparatopic antibody. At the same time, the mixture of pertuzumab and 3_17ET Fcab was not active in reducing the proliferation of SK-BR-3 cells: the reason can be on one hand about a 10-fold lower affinity of the Fcab to HER2 compared with trastuzumab, and the fact that their epitopes are overlapping, but not identical.

The intracellular fate of HER2 upon trastuzumab treatment has been interpreted as HER2 degradation [40] or also recycling [41]. A systematic study of several antibody-treated HER2-expressing cell lines concludes that in the highly expressing ones, the HER2 recycling is efficient and the surface levels only minimally reduced [42], and a similar effect was found for pertuzumab treatment [43]. In contrast, bivalent Fab fragments recognizing other epitopes, in particular the area between subdomain I and subdomain II opposite of the binding site of pertuzumab, can cause the oligomerization, internalization, and degradation of HER2 [44]. Other reports describe the reduction in HER2 levels on the cell surface following the activity of biparatopic constructs, such as in case of the SH3 domain reactive with subdomain I of HER2, fused with pertuzumab (COVA208) [43]. It is not likely that the surface reduction in HER2 is a consequence of the inhibition of protein synthesis, as it is described as a relatively stable protein whose surface levels are not affected by the cycloheximide-mediated inhibition of the protein synthesis until 16 h after treatment [45]. We observed a potentiated internalization of the biparatopic antibody after 3 and 12 h of incubation, and also this phenomenon can contribute to the faster internalization of HER2. However, as described previously for biparatopic constructs, the potent internalization of HER2 can be caused by the cross-linking and formation of larger receptor complexes than tetramers that can form after treatment with monoclonal antibodies alone [46]. In the case of the bispecific construct described here, the simultaneous binding to subdomain II and subdomain IV of HER2 can induce such extended oligomerization, through the engagement of all four binding sites. HER2 is usually considered to be endocytosis-resistant, and also targeting antibodies are not strongly internalized and rapidly recycled [41,47]. HER2 clustering has been long recognized as a method for overcoming the resistance to internalization and therefore addressed with several different strategies [48,49,50]. The endocytosis resistance of HER2 is believed to be based on its stabilizing interaction with heat-shock protein 90 (Hsp90), and HER2 internalization and degradation can be induced by Hsp90 inhibitors. HER2 can also be internalized upon activation of protein kinase C, but contrary to the anti-Hsp90 agents, this mediates a clathrin-independent internalization pathway without HER2 degradation [51]. Importantly, the cytotoxic mechanism by which the studied bispecific construct exerts its anti-proliferative activity is the induction of the apoptosis of HER2-positive cells, as demonstrated with the monitoring of the percentage of AnnexinV-positive cells and cells exhibiting Caspase 3/7 activity.

To conclude, the presented mAb^2^ appears a potent anti-HER2 agent, despite the moderate affinity of the Fcab-part for HER2. It would be interesting to study if an affinity-improved variant could display a more potent antiproliferative effect or possibly deplete surface HER2 faster. Recently, modified C_H_3 domains with antigen-binding properties were shown to be able to replace the C_H_1/C_L_ domain pair in an antibody [35], providing on one hand a different spatial constellation with respect to the Fab-accommodated antigen binding site, and on the other hand also increased the multiplicity of available binding sites for the second antigen. It was already shown that higher-valency conjugates with a similar binding affinity accelerate the depletion of surface HER2 [52], and therefore the investigations into such alternative antibody formats could as well deliver even more efficient anti-HER2 therapeutic candidates.

## Data Availability

The data presented in this study are available within this article or its supplementary material.

## Supplementary Materials

The following are available online at www.mdpi.com/2075-1729/11/11/1157/s1, Figure S1: Characterization of anti-HER2 Fcab 3_17ET, Table S1: Sequences of oligonucleotides used for mutagenesis and PCR amplification, Table S2: Amino acid sequences of constructs used in this study, Supplementary File S1: Supplementary Methods.
