# Supplementary material for: A Tetravalent Biparatopic Antibody Causes Strong HER2 Internalization and Inhibits Cellular Proliferation
Source: Life (Basel). 2021 Oct 29;11(11):1157. doi: 10.3390/life11111157 (PMC8624325; doi:10.3390/life11111157)
Supplement: Supplementary file 1 [file life-11-01157-s001.zip › life-1345996-supplementary.pdf]

life

MDPI

Supplementary materials of:

A Tetravalent Biparatopic Antibody Causes Strong HER2 Internalization and Inhibits Cellular Proliferation

Filippo Benedetti, Katharina Stadlbauer, Gerhard Stadlmayr, Florian Rüker and Gordana Wozniak-Knopp *

Christian Doppler Laboratory for Innovative Immunotherapeutics, Institute of Molecular Biotechnology, Department of Biotechnology, University of Natural Resources and Life Sciences, Vienna (BOKU), Muthgasse 18, 1190 Vienna, Austria; Filippo.benedetti@boku.ac.at (F.B.); katharina.stadlbauer@boku.ac.at (K.S.); Gerhard.stadlmayr@boku.ac.at (G.S.); florian.rueker@boku.ac.at (F.R.)

* Correspondence: gordana.wozniak@boku.ac.at; Tel.: +43-1-47654-79868

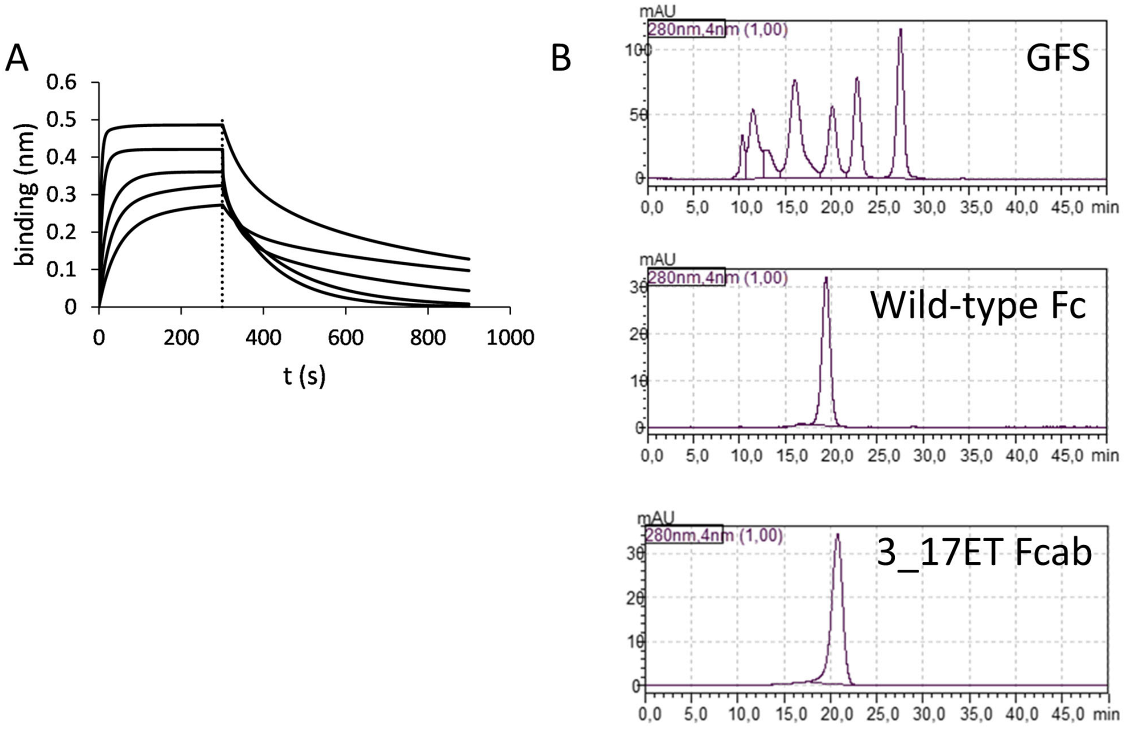

Figure 1. Characterization of anti-HER2 Fcab 3_17ET. (**A**) Binding to biotinylated extracellular domain of HER2 measured in a biolayer interferometry experiment; (**B**) HPLC-SEC of wild-type Fc and 3_17ET Fcab. Gel filtration standard (GFS) included 670, 158, 44, 17 and 1.35 kDa-proteins (Bio-Rad).

Table S1. Sequences of oligonucleotides used for mutagenesis and PCR amplification.

Oligonucleotide	Oligonucleotide Sequence (5′-3′)
	**Mutagenesis of 3_17 Fcab (R415bE)**
3_17E	gtggcgcgac acagcgagac tatgtggagg tgggggcacg
3_17Ea	cgtgccccca cctccacata gtctcgctgt gtcgcgccac
	**Cloning of mutated CH3 domain**
ch3xho1	gaaactcgag aaccacaggt gtacaccctg
ch3sbam2	gattggatcc tcatttaccc ggagacagg

Table S2. Amino acid sequences of constructs used in this study. Residues mutated in the CH3 domain are in bold (AB loop: red, CD loop: green, EF loop: blue, C-terminus: orange), sequences of pertuzumab Fab fragment are green and trastuzumab Fab fragment purple.

	Amino Acid Sequence
Fc Fragments	
**Wild-type Fc**	TCPPCPAPELLGGPSVFLFPPKPKDTLMISRTPEVTCVVVDVSHEDPEVKFNWY-VDGVEVHNAKTKPREEQYNSTYRVVSVLTVLHQDWLNGKEYKCKVSNKALPAPIEKTISKAKGQPREPQVYTLPPSRDELTKNQVSLTCLVKGFYPSDIAVEWESNGQPENNYKTTPPVLDSDGS-FFLYSKLTVDKSRWQQGNVFSCSVMHEALHNHYTQKSLSLSPGK
**3_17ET Fcab**	TCPPCPAPELLGGPSVFLFPPKPKDTLMISRTPEVTCVVVDVSHEDPEVKFNWY-VDGVEVHNAKTKPREEQYNSTYRVVSVLTVLHQDWLNGKEYKCKVSNKALPAPIEKTISKAKGQPREPQVYTLPPSRDE**YLHGD**VSLTCLVKGFYPSDIAVEWESNGQPENNYKTTPPVLDSDGS-FFLYSKLTV**ARHSETMW**RW**GH**GNVFTCSVMHEALHNHYTQKSLSLSPGK
Antibodies	
**Pertuzumab**	
Light chain	DIQMTQSPSSLSASVGDRVTITCKASQDVSIGVAWYQQKPGKAP-KLLIYSASYRYTGVPSRFSGSGSGTDFTLTISSLQPEDFATYYCQQYYIYPYTFGQGTKVEIKRTVAAPSVFIFPPSDEQLKSGTASVVCLLNNFYPREAKVQWKVDNALQSGNSQESVTEQDSKDSTYSLSSTLT-LSKADYEKHKVYACEVTHQGLSSPVTKSFNRGEC
Wild-type heavy chain	EVQLVESGGGLVQPGGSLRLSCAASGFTFTDYTMDWVRQAPGKGLEWVADVNPNSGG-SIYNQRFKGRFTLSVDRSKNTLYLQMNSLRAEDTAVYYCARNLGPSFYFDYWGQGTLVTVSSASTKGPSVFPLAPSSKSTSGGTAALGCLVKDYFPEPVTVSWNSGALTSGVHTFPAVLQSSGLYS-LSSVVTVPSSSLGTQTYICNVNHKPSNTKVDKKVEPKSCDKTHTCPPCPAPELLGGPSVFLFPPKPKDTLMISRTPEVTCVVVDVSHEDPEVKFNWYVDGVEVHNAK-TKPREEQYNSTYRVVSVLTVLHQDWLNGKEYKCKVSNKALPAPIEKTISKAKGQPREPQVYTLPPSRDELTKNQVSLTCLVKGFYPSDIAVEWESNGQPENNYKTTPPVLDSDGS-FFLYSKLTVDKSRWQQGNVFSCSVMHEALHNHYTQKSLSLSPGK
Pertuzumab_3_17ET mAb²	EVQLVESGGGLVQPGGSLRLSCAASGFTFTDYTMDWVRQAPGKGLEWVADVNPNSGG-SIYNQRFKGRFTLSVDRSKNTLYLQMNSLRAEDTAVYYCARNLGPSFYFDYWGQGTLVTVSSASTKGPSVFPLAPSSKSTSGGTAALGCLVKDYFPEPVTVSWNSGALTSGVHTFPAVLQSSGLYS-LSSVVTVPSSSLGTQTYICNVNHKPSNTKVDKKVEPKSCDKTHTCPPCPAPELLGGPSVFLFPPKPKDTLMISRTPEVTCVVVDVSHEDPEVKFNWYVDGVEVHNAK-TKPREEQYNSTYRVVSVLTVLHQDWLNGKEYKCKVSNKALPAPIEKTISKAKGQPREPQVYTLPPSRDE**YLHGD**VSLTCLVKGFYPSDIAVEWESNGQPENNYKTTPPVLDSDGSFFLYSKLT-V**ARHSETMW**RW**GH**GNVFTCSVMHEALHNHYTQKSLSLSPGK
**Trastuzumab**	
Light chain	DIQMTQSPSSLSASVGDRVTITCRASQDVNTAVAWYQQKPGKAP-KLLIYSASFLYSGVPSRFSGSRSGTDFTLTISSLQPEDFATYYCQQHYTTPPTFGQGTKVEIKRTVAAPSVFIFPPSDEQLKSGTASVVCLLNNFYPREAKVQWKVDNALQSGNSQESVTEQDSKDSTYSLSSTLT-LSKADYEKHKVYACEVTHQGLSSPVTKSFNRGEC
Wild-type heavy chain	EVQLVESGGGLVQPGGSLRLSCAASGFNIKDTYIHWVRQAPGKGLEWVARIYPTNGYTRYADSVKGRFTISADTSKNTAYLQMNSLRAEDTAVYYCSRWGGDGFYAMDYWGQGTLVTVSSASTKGPSVFPLAPSSKSTSGGTAALGCLVKDYFPEPVTVSWNSGALTSGVHTFPAVLQSSGLYSLSSVVTVPSSSLGTQTYICNVNHKPSNTKVDKKVEPKSCDKTHTCPPCPAPELLGGPSVFLFPPKPKDTLMISRTPEVTCVVVDVSHEDPEVKFNWYVDGVEVHNAKTKPREEQYNSTYRVVSVLTVLHQDWLNGKEYKCKVSNKALPAPIEKTISKAKGQPREPQVYTLPPSRDELMTKNQVSLTCLVKGFYPSDIAVEWESNGQPENNYKTTPPVLDSDGSFFLYSKLTVDKSRWQQGNVFSCSVMHEALHNHYTQKSLSLSPGK
Trastuzumab_CT6 mAb²	EVQLVESGGGLVQPGGSLRLSCAASGFNIKDTYIHWVRQAPGKGLEWVARIYPTNGYTRYADSVKGRFTISADTSKNTAYLQMNSLRAEDTAVYYCSRWGGDGFYAMDYWGQGTLVTVSSASTKGPSVFPLAPSSKSTSGGTAALGCLVKDYFPEPVTVSWNSGALTSGVHTFPAVLQSSGLYSLSSVVTVPSSSLGTQTYICNVNHKPSNTKVDKKVEPKSCDKTHTCPPCPAPELLGGPSVFLFPPKPKDTLMISRTPEVTCVVVDVSHEDPEVKFNWYVDGVEVHNAKTKPREEQYNSTYRVVSVLTVLHQDWLNGKEYKCKVSNKALPAPIEKTISKAKGQPREPQVYTLPPSRDEL**RFY**QVSLTCLVKGFYPSDIAVEWESNGQP**DIFPNGL**NYKTTPPVLDSDGSFFLYSKLTV**PYPS**WL**MGT** **R**FSCSVMHEALHNHYTQK**HLEYQWPT**

Supplementary File S1

Supplementary Methods

Biolayer interferometry (BLI).

Binding of Fcab 3_17 to Her2 was measured using BLI. Her2 ECD-Fc (SinoBiological) was reconstituted at 250 μg/mL and biotinylated using EZ-Link NHS-LC-LC-Biotin (Thermo Fisher Scientific) exactly according to manufacturer's instructions with biotin to protein molar ratio of 3:1, followed by dialysis against 100-fold volume of PBS overnight at 4 °C using Snakeskin dialysis tubing with 10000 Da MWCO (Pierce, Thermo Fisher Scientific). The kinetic parameters of Her2 binding at 25 °C were determined with Octet RED96e system (ForteBio, Molecular Devices). Streptavidin tips, equilibrated in PBS with Kinetic Buffer (ForteBio, Molecular Devices) were loaded with 5 μg/mL biotinylated Her2 ECD-Fc for 300 s with agitation at 1000 rpm. Fcab in 2-fold serial dilutions starting from 1800 nM interacted with the antigen for 300 s and were let to dissociate into PBS with Kinetic Buffer buffer for 600 s. Sensorgrams of antibody dilutions binding to non-coated tips and Her2-coated tip immersed into the assay buffer in the association and dissociation step were subtracted as background before fitting the data to derive kinetic parameters with ForteBio Analysis software version 11.0.High performance liquid chromatography-size exclusion chromatography (HPLC-SEC) for Fcabs.

A Shimadzu LC-20A Prominence system equipped with a diode array detector and a refractive index detector was used with a TSK G3000SWXL column (7.8630 mm). Chromatography was conducted with phosphate buffered saline (PBS) with 200 mM NaCl as the mobile phase buffer with a constant flow rate of 1 mL/min. A total of 25 μg protein at about 1 mg/mL were loaded on the column for analysis. A set of molecular weight standards ranging from 670 to 1.3 kDa (Bio-RAD) was used as gel filtration standard.
